# Protein Kinase CK2 represents a new target to boost Ibrutinib and Venetoclax induced cytotoxicity in mantle cell lymphoma

**DOI:** 10.3389/fcell.2022.935023

**Published:** 2022-08-11

**Authors:** Sabrina Manni, Maria Pesavento, Zaira Spinello, Lara Saggin, Arash Arjomand, Anna Fregnani, Laura Quotti Tubi, Greta Scapinello, Carmela Gurrieri, Gianpietro Semenzato, Livio Trentin, Francesco Piazza

**Affiliations:** ^1^ Department of Medicine-DIMED, Hematology and Clinical Immunology Section, University of Padova, Padova, Italy; ^2^ Myeloma and Lymphoma Pathobiology Lab, Veneto Institute of Molecular Medicine, Padova, Italy

**Keywords:** mantle cell lymphoma, CK2, ibrutinib, venetoclax, target therapy

## Abstract

Mantle cell lymphoma (MCL) is an incurable B cell non-Hodgkin lymphoma, characterized by frequent relapses. In the last decade, the pro-survival pathways related to BCR signaling and Bcl-2 have been considered rational therapeutic targets in B cell derived lymphomas. The BTK inhibitor Ibrutinib and the Bcl-2 inhibitor Venetoclax are emerging as effective drugs for MCL. However, primary and acquired resistance also to these agents may occur. Protein Kinase CK2 is a S/T kinase overexpressed in many solid and blood-derived tumours. CK2 promotes cancer cell growth and clonal expansion, sustaining pivotal survival signaling cascades, such as the ones dependent on AKT, NF-κB, STAT3 and others, counteracting apoptosis through a “non-oncogene” addiction mechanism. We previously showed that CK2 is overexpressed in MCL and regulates the levels of activating phosphorylation on S529 of the NF-κB family member p65/RelA. In the present study, we investigated the effects of CK2 inactivation on MCL cell proliferation, survival and apoptosis and this kinase’s involvement in the BCR and Bcl-2 related signaling. By employing CK2 loss of function MCL cell models, we demonstrated that CK2 sustains BCR signaling (such as BTK, NF-κB and AKT) and the Bcl-2-related Mcl-1 expression. CK2 inactivation enhanced Ibrutinib and Venetoclax-induced cytotoxicity. The demonstration of a CK2-dependent upregulation of pathways that may antagonize the effect of these drugs may offer a novel strategy to overcome primary and secondary resistance.

## Introduction

Mantle cell lymphoma (MCL) accounts for about 3–10% of all B-cell non-Hodgkin lymphomas (NHL) and is characterized by the propensity to frequent relapses and poor prognosis (Jain and Wang, 2022).

B-Cell Receptor (BCR)-linked survival signaling cascades, such as Phosphatidylinositide 3-Kinases/AKT (PI3K/AKT), mammalian Target Of Rapamycin (mTOR), Nuclear Factor kappa-light-chain-enhancer of activated B cells (NF-kB), the Extracellular signal-Regulated Kinase (ERK) cascades and the Bcl-2 family of apoptosis regulators, are chronic active in MCL and enhance tumor proliferation, facilitating evasion of apoptosis. BCR signaling is essential for the development and function of B lymphocytes and is a key molecule for the growth of many B-NHL. In the last decade, the pro-survival pathways relying on BCR signaling and Bcl-2 family proteins have emerged as rational therapeutic targets. Chemical inhibitors directed against BCR signaling kinases, such as Ibrutinib and Acalabrutinib (BTK) and Idelalisib and Duvelisib (PI3K), have been successfully used in the therapy of B cell malignancies (Profitós-Pelejà et al., 2022). Ibrutinib is the first-in-class Bruton Tyrosine Kinase (BTK) inhibitor which acts switching off BTK-dependent BCR signaling and is currently approved for relapsed/refractory (R/R) MCL. Venetoclax is a first-in class selective Bcl-2 inhibitor with demonstrated clinical activity in MCL, in particular in combination with Ibrutinib (Davids et al., 2017; Tam et al., 2018). However, primary and acquired resistance to Ibrutinib, mainly due to BTK C481 or PLCγ mutations (Hershkovitz-Rokah et al., 2018), and to Venetoclax, mainly due to upregulation of other pro-survival Bcl-2 family members, may occur (Huang et al., 2022; Jain and Wang, 2022). Therefore, even with the introduction of these novel drugs, the development of refractoriness and resistance is still the main unsolved clinical-biological issue.

CK2 is a S/T kinase, composed by a tetramer of two catalytic CK2α (most represented) or CK2α’ and two regulatory CK2β subunits. CK2 promotes cancer cell proliferation and tumor progression by antagonizing extrinsic and intrinsic apoptosis in a “non-oncogene” addiction fashion (Mandato et al., 2016; Spinello et al., 2021). CK2 also regulates cellular processes central for cancer biology (p53, Wnt/β-catenin, AKT and NF-kB survival signaling pathways) (Spinello et al., 2021). CK2 phosphorylates BH3-Interacting Domain Death Agonist (BID) on T58, preventing its cleavage from caspase 8, protecting from apoptosis (Hellwig et al., 2010). By sustaining AKT function (Di Maira et al., 2009), CK2 contributes to BAD inactivation. CK2 also phosphorylates Apoptosis Repressor with Caspase recruitment domain (ARC) at T149 critically supporting caspase inhibition (Li et al., 2002). Lastly, being the CK2 recognition motif strikingly similar to that of caspases, the CK2-dependent phosphorylation of caspases substrates protects them from the proteolytic cleavage (Duncan et al., 2011). We previously showed that CK2 sustains tumor progression in multiple myeloma (MM), NHL, leukemias and other haematological malignancies, being CK2 overexpressed in B cell derived tumors such as MM and MCL (Manni et al., 2013), follicular lymphoma and diffuse large B cell lymphoma (DLBCL) (Pizzi et al., 2015). Moreover CK2 regulates the levels of pro-survival activating p65 NF-κB S529 and STAT3 S727 phosphorylations in MCL (Manni et al., 2013). More recently, we demonstrated that CK2 regulates BCR signaling in DLBCL cells since its inhibition is associated to impairment of Ca^2+^ mobilization and of phosphorylation of NF-κB and AKT (Pizzi et al., 2015; Mandato et al., 2018). Phosphorylated AKT on S129 (Di Maira et al., 2009) and p65 NF-κB on S529 (Wang et al., 2000) are direct CK2 targets important for the activity of these two BCR-activated pro-survival molecules. Different CK2 inhibitors have been developed, and the ATP competitive, clinical-grade CK2 inhibitor CX-4945 (Silmitasertib) (Siddiqui-Jain et al., 2010) is already used in Phase I/II clinical trial in solid tumors and R/R MM (ClinicalTrials.gov Identifier NCT01199718), and very recently in Phase II clinical trial for severe coronavirus disease 2019 -COVID 19- (ClinicalTrials.gov Identifier: NCT04668209).

Given the key role of CK2 in signaling pathways that could sustain active BCR and Bcl-2 family related cascades in MCL, we investigated this kinase’s involvement in the BCR signaling and the effects of its inactivation on MCL cell proliferation and survival. We also evaluated if CK2 inactivation could enhance Ibrutinib and Venetoclax-induced cytotoxicity offering a potential strategy to overcome primary and secondary resistance to these drugs.

## Materials and methods

**Patients and cell cultures**. Jeko-1, Rec-1, Granta-519 MCL cell lines, and primary MCL B cells were isolated and cultured as previously described (Manni et al., 2021). Human derived B cells were obtained after achieving informed consent according to the declaration of Helsinki and upon approval by the Ethic Committee of the Padova University Hospital Internal Institutional Board (protocol # 4,089/AO/17).

**Chemicals.** Ibrutinib, Venetoclax, Bortezomib and Z-VAD-FMK were from Selleck chemicals (United States); IPTG was from MERCK (Italy); CX-4945 was from Activate Scientific (Germany).

**Evaluation of apoptosis.** Apoptosis was assessed by Annexin V/Propidium Iodide (PI) staining (IMMUNOSTEP, Spain) and FACS analysis performed as in (Manni et al., 2017).

**Cell cycle analysis**. It was performed as described in (Manni et al., 2017).

**Assessment of drug concentration-effect and calculation of the combination index (CI)**. Jeko-1, Rec-1 and Granta-519 cells were plated into 96 well plates in 100 μL of medium. CX-4945, Ibrutinib, and Venetoclax were added at different concentrations for 72 h alone or in combination. Cell viability was analyzed with 3-(4,5-dimethylthiazol-2-yl)-2,5-diphenyltetrazolium bromide (MTT) assay and the CI was calculated as performed in (Manni et al., 2012).

**Western blot (WB).** WB was performed as described in (Piazza et al., 2010). Antibodies used were: CK2α, CK2β, S129 AKT (abcam, United Kingdom), Mcl-1, S473 AKT, Y223 BTK, total BTK, PARP, S536 p65 NF-kB (Cell signaling Technology, MA, United States); S529 p65 NF-κB (Santa Cruz Biotechnology, Inc.; Italy), GAPDH (Millipore, Italy), β-actin (Sigma-Aldrich, Italy); Caspase 3 (Enzo Life Science, United Kingdom). Images were acquired using the Image Quant LAS 500 chemiluminescence detection system (GE Healthcare, United States) and densitometry of the bands was performed using the ImageQuantTL software.

**RNA interference.** We generated inducible CK2α or CK2β directed shRNA IPTG inducible MCL cell clones performing shRNA lentiviral transduction. Jeko-1 cells were transduced with the IPTG inducible lentiviral particles carrying *CSNK2A1*-specific shRNA (pLKO_IPTG_3XLacO, Sigma-Aldrich, Italy) with the sequence code TRCN0000320858 or *CSNK2B* directed shRNA (sequence code TRCN0000003796). For cell transduction 2 × 10^4^ cells were infected with a multiplicity of infection of 10, using the spinfection method (45 min, 1,000 rpm at 32°C), in the presence of 8 μg/ml polybrene (Sigma-Aldrich, Italy). Puromycin selection (1 μg/ml) was initiated 2 days after transduction. To induce CK2α or CK2β silencing, cells were incubated with IPTG 500 µM every 2 days for a total of 6 days of silencing, time lapse in which the best knockdown efficacy was obtained.

**CK2α silencing *in vivo* in a human MCL xenograft murine model.** NOD SCID mice were purchased from Envigo. All animal studies were conducted according to protocol # 160/2017 PR, approved by the Italian Ministry of Health. 3 × 10^6^ CK2α shRNA Jeko-1 and Jeko-1 WT cells were injected subcutaneously in 100 µL of a mix of RPMI: matrigel matrix 10 mg/ml (Corning, United States, cat number 354263) (1:1 ratio) on the right and on the left flank respectively of NOD SCID mice. Once the tumor reached a measurable size (around 0.245 cm^3^, approximately after 10 days of injection), mice were divided into two groups (control/untreated and IPTG treated). Four mice for each condition were used. IPTG was administered at the concentration of 20 mM intraperitoneally (IP) (in 200 µL of physiological solution 0.9% NaCl every other day) and at 4 mM in drinking water (refreshing it twice a week) for 25 days. Control mice were injected IP with 200 µL physiological solution 0.9% NaCl every other day. Tumors were then dissected, weighed, and cut into pieces for protein analysis as per standard protocols.

**Mitochondrial membrane potential (MMP) measurement.** The MMP was measured by JC-10 dye (Sigma Aldrich, # number MAK160) staining. JC-10 forms reversible red-fluorescent aggregates (*λ*_ex_ = 540/*λ*_em_ = 590 nm) in healthy mitochondria of cells. MMP reduction during apoptosis caused a failure to maintain JC-10 in the mitochondria and an increase of its monomeric, green, fluorescent form (*λ*_ex_ = 490/*λ*_em_ = 525 nm). Cells were stained following the manufacturer’s instructions and analysed using flow cytometry (FACS Canto II Cell Cytometer and FACS Diva Software, BD-Beckton-Dickinson, Italy).

**Quantitative real-time PCR.** Performed as in (Manni et al., 2021) using the QuantStudio five detection system (Applied Biosystem, CA, United States) with the QuantStudioTM Design and Analysis Software v.1.4.3. The primers used are the following:

Bcl-2 Forward 5′-3′ TGT​GGA​TGA​CTG​AGT​ACC​TGA​ACC and Reverse 5′-3′ AAA​GGC​ATC​CCA​GCC​TCC; BCL2L1 (BCL-XL) Forward 5′-3′ GCA​GGT​ATT​GGT​GAG​TCG​GAT​CGC and Reverse 5′-3′ CAC​AAA​AGT​ATC​CCA​GCC​GCC; CSNK2A1 Forward 5′-3′ TCA​TGA​GCA​CAG​AAA​GCT​ACG​A and Reverse 5′-3′ AAT​GGC​TCC​TTC​CGA​AAG​ATC; IL-6 Forward 5′-3′ GGC​ACT​GGC​AGA​AAA​CAA​CCT​G and Reverse 5′-3′ TCA​CCA​GGC​AAG​TCT​CCT​CAT​TGA​AT; Mcl-1 Forward 5′-3′ GAA​AGT​ATC​ACA​GAC​GTT​CTC​GTA​AGG and Reverse 5′-3′ AAC​CCA​TCC​CAG​CCT​CTT​TG.

GAPDH Forward 5′-3′ AAT​GGA​AAT​CCC​ATC​ACC​ATC​T and Reverse 5′-3′ CGC​CCC​ACT​TGA​TTT​TGG.

**Statistical analysis.** Data were analyzed for statistical significance with the two-tail unpaired Student’s *t* test or ANOVA analysis of variance with *post-hoc* corrections and statistical significance was considered with *p* values below 0.05.

## Results

### CK2α sustains MCL cell survival through the activation of active BCR-linked survival signaling

**FIGURE 1:**
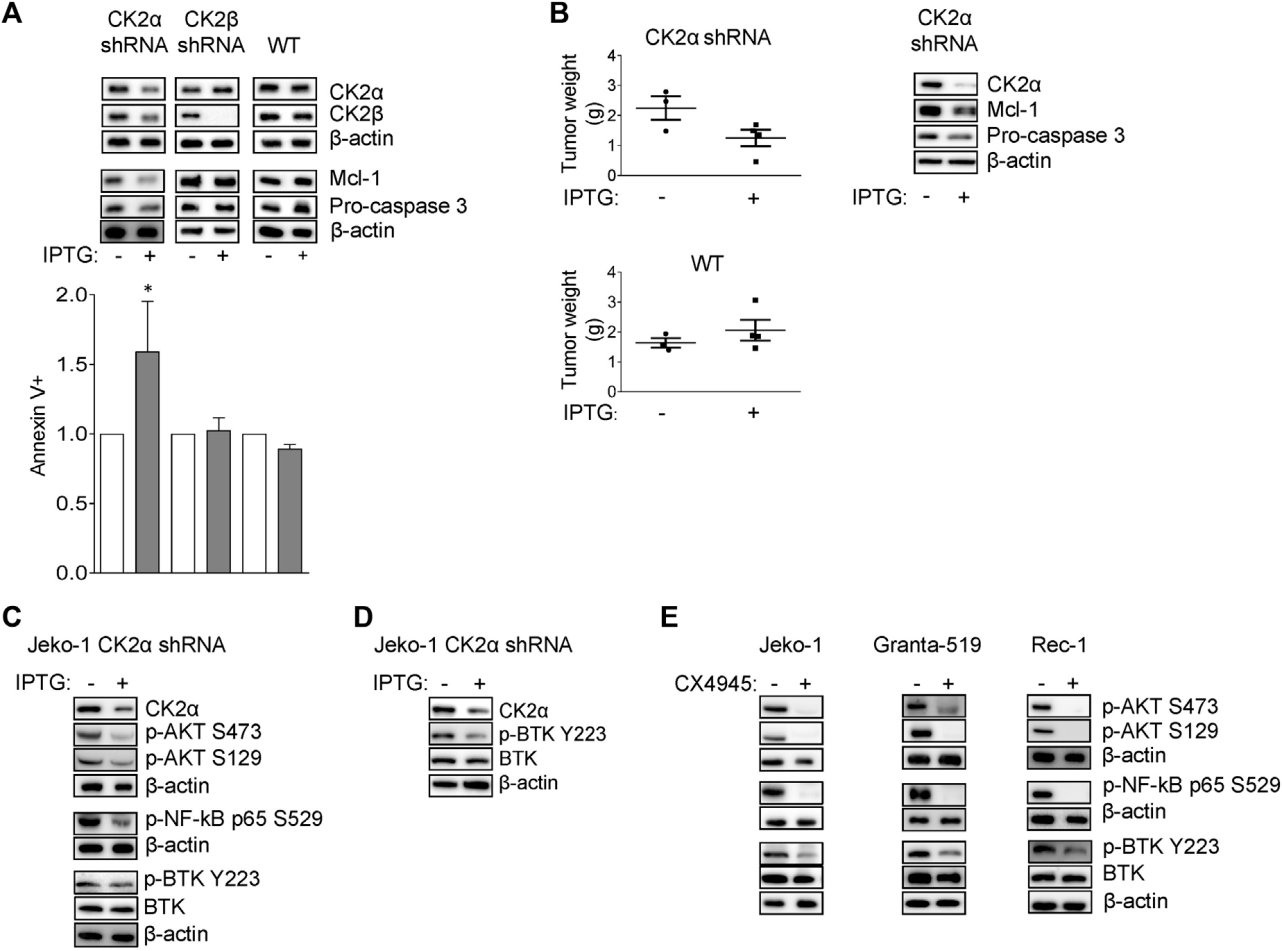
CK2α sustains MCL cell growth and survival signaling pathways downstream the BCR. **(A)** Representative WB (upper panel) of CK2α, CK2β, Mcl-1 and Pro-caspase 3 expression and histogram showing the percentage of Annexin V positive Jeko-1 CK2α shRNA, Jeko-1 CK2β shRNA and Jeko-1 WT cells (lower panel) after 6 days treatment with IPTG 500 μM. β-actin was used as loading control. Data are expressed as ratio over untreated cells, mean ± SD of *n* = 8 (Jeko-1 CK2α and CK2β shRNA) and *n* = 4 (Jeko-1 WT) independent experiments. * indicates *p* < 0.05 compared to untreated cells. **(B)** Tumor weight (grams, left panel) of Jeko-1 CK2α shRNA and Jeko-1 WT cells xenografted in NOD SCID mice and treated *in vivo* with IPTG for 25 days. Data are expressed as mean ± SD. The number of mice is indicated by the symbols. Representative WB (right panel) showing *in vivo* CK2α knock down efficacy and Mcl-1 and Pro-caspase3 reduction. β-actin was used as loading control. **(C)** Representative WB of NF-ĸB, PI3K/AKT and BTK dependent signaling in Jeko-1 CK2α shRNA cells after 6 days induction with IPTG 500 μM. **(D)** Representative WB showing the expression levels of phosphorylated BTK on Y223 (p-BTK Y223) *in vivo* in Jeko-1 CK2α shRNA cells xenografted, and treated with IPTG as in **(B)**. **(E)** Representative WB of NF-ĸB, PI3K/AKT and BTK dependent signaling in MCL cell lines Jeko-1, Granta-519 and Rec-1 after 24 h treatment with DMSO (not higher than 0.1% v/v) or CX-4945 (2.5 μM for Jeko-1 and Granta-519; 0.5 μM for Rec-1). β-actin was used as loading control. Experiments were repeated at least three times for each cell line.

To specifically assess the contribution of each CK2 subunit to MCL cell growth we generated Jeko-1 MCL cells stably transduced with lentiviral vectors, expressing a shRNA against CK2α or CK2β in an Isopropyl-b-D-1-thiogalattopiranoside (IPTG)-inducible manner. Upon 6 days of IPTG treatment, we confirmed efficient CK2α or CK2β protein silencing (Figure 1A). No off-target effects of IPTG *per se* were seen, as CK2α/β protein levels remained unchanged in IPTG-treated Jeko-1 wild-type (WT) cells. Remarkably, Annexin V (AV) and Propidium Iodide (PI) labelling and FACS analysis revealed a substantial induction of apoptosis in CK2α but not CK2β-silenced cells, which was confirmed by the reduction of the anti-apoptotic Mcl-1 and Pro-caspase 3 proteins (Figure 1A and Supplementary Figure S1). As control, to exclude IPTG off-target induced toxicity, we treated Jeko-1 WT cells with IPTG for the same amount of time. As expected, IPTG-treated WT cells remained viable and did not show any difference in the expression level of Mcl-1 and Pro-caspase 3 proteins, confirming the efficacy/specificity of the silencing strategy (Figure 1A and Supplementary Figure S1). We next performed *in vivo* xenotransplant experiments by implanting CK2α shRNA Jeko-1 cells in the flank of NOD-SCID mice and treating them with intraperitoneal IPTG for as long as 25 days. CK2α silencing *in vivo* was efficient (Figure 1B and Supplementary Figure S1) and associated to a trend towards a reduction in the weight of tumors, which displayed a drop in the expression of Mcl-1 and Pro-caspase 3 anti-apoptotic proteins. As a control, Jeko-1 WT cells grew to the same extent in IPTG-treated or untreated mice (Figure 1B). Next, we analysed CK2α contribution in the regulation of pro-survival signaling pathways downstream from the BCR. First, we evaluated the expression levels of key molecules involved in the NF-κB, PI3K/AKT and BTK signaling in cultured Jeko-1 CK2α shRNA cell line. A reduction of the activating phosphorylation levels of p65/RelA on S529 (Figure 1C and Supplementary Figure S1) and consequently of the mRNA expression level of the NF-κB targets *BCL2L1* (which encodes for the BCL-XL protein)*, Bcl-2* and *IL-6* (Supplementary Figure S2), and of AKT on S129 and S473 was seen in CK2α-silenced cells (Figure 1C and Supplementary Figure S1). Intriguingly, we also observed a reduction in the levels of phosphorylated BTK on Y223 in cultured cells (Figure 1C and Supplementary Figure S1) and *in vivo* in mouse xenografted IPTG-treated CK2α shRNA-expressing Jeko-1 cells (Figure 1D and Supplementary Figure S1).

To further validate the role of CK2α in BCR signaling, we treated Jeko-1, Granta-519 and Rec-1 cells with the clinical-grade CK2 inhibitor CX-4945 for 24 h. A reduction of the phosphorylation levels of p65/RelA, AKT and BTK were observed also in these conditions (Figure 1E and Supplementary Figure S1), suggesting that CK2α acts on NF-κB, PI3K/AKT and BTK signaling, affecting the spectrum of cascades downstream from the BCR.

### CK2 inactivation boosts Ibrutinib-induced cytotoxicity in MCL cells

**FIGURE 2:**
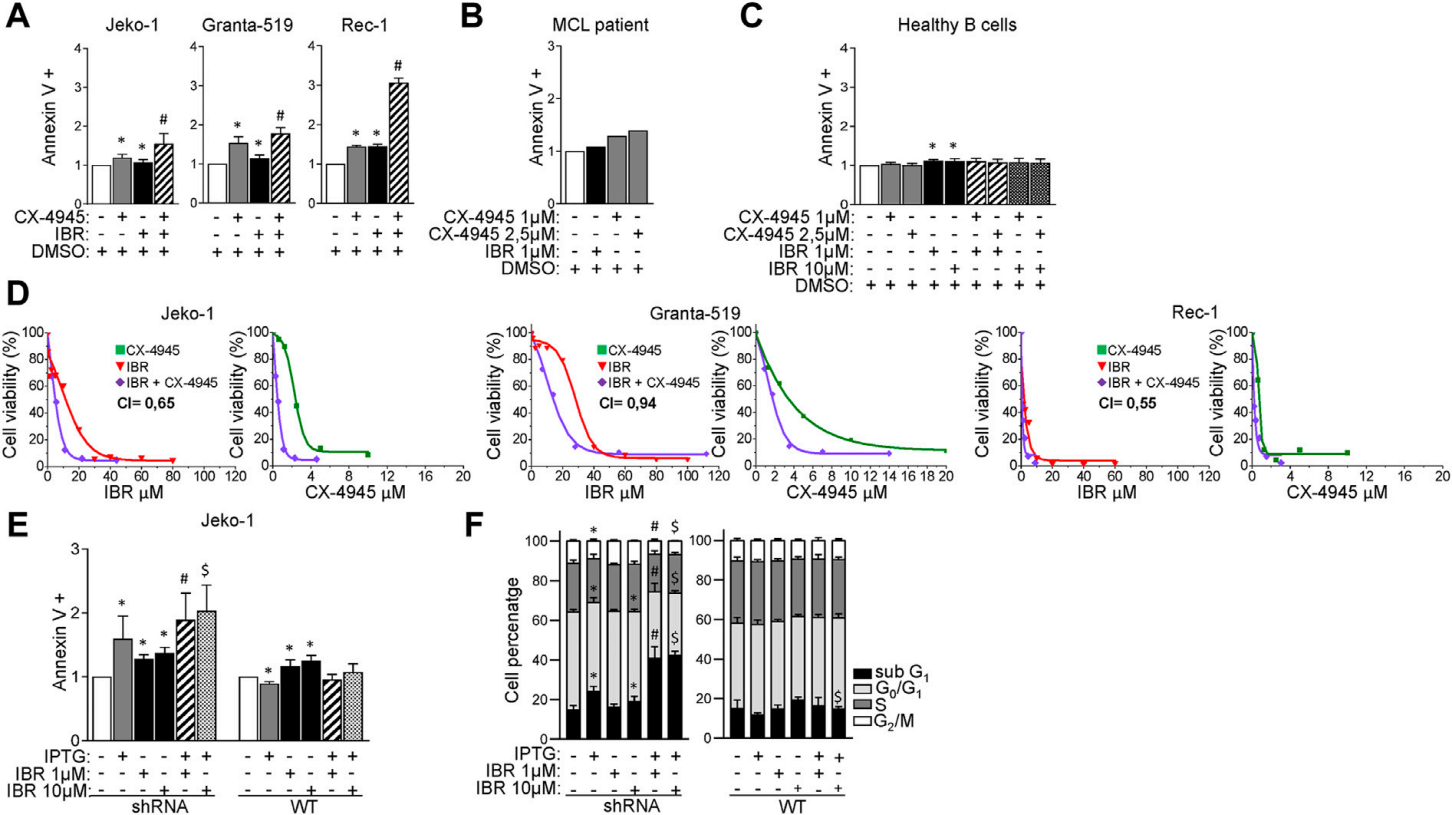
CK2α inhibition boosts Ibrutinib induced cytotoxicity. **(A)** Histograms showing the percentage of Annexin V positive Jeko-1, Granta-519 and Rec-1 cells after 24 h treatment with DMSO (not higher than 0.1% v/v; white column), CX-4945 (1 μM for Jeko-1 and Rec-1, 2.5 μM for Granta-519; grey columns), IBR (1 μM for each cell line; black columns) or their combination (striped columns). Data are expressed as ratio over DMSO treated cells, mean ± SD of *n* = 6 (Jeko-1), *n* = 5 (Granta-519) and *n* = 3 (Rec-1) independent experiments. * indicates *p* < 0.05 compared to DMSO; # indicates *p* < 0.05 compared to both CX-4945 and IBR only treated cells. **(B)** Histogram showing the percentage of AV positive B cells derived from one Ibrutinib resistant MCL patient after 24 h treatment with DMSO (not higher than 0.1% v/v; white column), Ibrutinib (1 μM; black column), CX-4945 (1 μM and 2.5 μM; grey columns). Data are expressed as ratio over DMSO treated cells. **(C)** Histogram depicting the percentage of Annexin V positive healthy B cells after 24 h treatment with DMSO (not higher than 0.1% v/v; white column), CX-4945 (1 μM and 2.5 μM; grey columns), IBR (1 and 10 μM; black columns) or their combination (striped and dotted columns). Data are expressed as ratio over DMSO treated cells, mean ± SD of *n* = 3 independent experiments. * indicates *p* < 0.05 compared to DMSO. **(D)** Dose response curves of Jeko-1, Granta-519 and Rec-1 cells incubated for 72 h with increasing concentrations of CX-4945 (green curves), Ibrutinib (red curves) or their combination (purple curves). Cell viability was assessed by MTT test. For Jeko-1, IC_50_ of the single treatments were 2.3 μM for CX-4945 and 11 μM for IBR. IC_50_ for CX-4945 used in combination with IBR was 0.5 μM, while IC_50_ for IBR used in association with CX-4945 was 4.9 μM. The calculated CI was 0.65. For Granta-519, IC_50_ of the single treatments were 3.5 μM for CX-4945 and 28 μM for IBR. IC_50_ for CX-4945 used in combination with IBR was 1.67 μM, while IC_50_ for IBR used in association with CX-4945 was 13.2 μM. The calculated CI was 0.94. For Rec-1, IC_50_ of the single treatments were 0.76 μM for CX-4945 and 2.26 μM for IBR. IC_50_ for CX-4945 used in combination with IBR was 0.21 μM, while IC_50_ for IBR used in association with CX-4945 was 0.63 μM. The calculated CI was 0.55. Data are presented as averaged percentage over control of three independent experiments for each cell line. **(E)** Histogram showing the percentage of Annexin V positive Jeko-1 CK2α shRNA and Jeko-1 WT cells after 6 days induction with IPTG 500 μM (grey column), 24 h treatment with IBR (1 and 10 μM, black columns) or their combination (stripped and dotted columns). Data are expressed as ratio over untreated cells, mean ± SD of *n* = 8 (Jeko-1 CK2α shRNA) and *n* = 4 (Jeko-1 WT) independent experiments. * indicates *p* < 0.05 compared to untreated cells; # indicates *p* < 0.05 compared to both IPTG and IBR 1 μM only treated cells; $ indicates *p* < 0.05 compared to both IPTG and IBR 10 μM only treated cells. **(F)** Cell cycle distribution of Jeko-1 CK2α shRNA and Jeko-1 WT cells treated as in **(E)**. Histograms columns sections represent mean ± SD of *n* = 4 independent experiments for each cell line of the sub G_1_ (black), G₀/G₁ (light grey), S (dark grey) and G₂/M (white) phases.* indicates *p* < 0.05 compared to untreated cells; # indicates *p* < 0.05 compared to both IPTG and IBR 1 μM only treated cells; $ indicates *p* < 0.05 compared to both IPTG and IBR 10 μM only treated cells.

We next sought to investigate whether CK2 inhibition could affect the responsiveness of MCL cells to Ibrutinib. To this aim, MCL cells were treated for 24 h with Ibrutinib, CX-4945 or their combination. For each cell line the association of BTK and CK2 inhibitors resulted in a significantly higher frequency of apoptotic cell death as compared to that induced by the single agents (Figure 2A). Of note, CX-4945 caused apoptosis also in B cells derived from one Ibrutinib-refractory patient, (Figure 2B). CX-4945 and Ibrutinib did not cooperate in inducing apoptosis in B-cells derived from healthy donors’ peripheral blood (Figure 2C) suggesting a lower sensitivity to these drugs of non-neoplastic B-cells as compared to the malignant ones. Three-(4,5-dimethylthiazol-2-yl)-2,5-diphenyltetrazolium bromide (MTT) viability assay was performed on Granta-519, Jeko-1 and Rec-1 cells treated with increasing concentrations of Ibrutinib, CX-4945 or their combination for 72 h. For each cell line, including Granta-519 (which is less sensitive to Ibrutinib as we previously published (Manni et al., 2021)), the calculated combination index (CI) was less than 1, indicating a synergistic effect between the two drugs (Figure 2D).

To exclude off-target effects of CX-4945, we repeated the experiments inactivating CK2α by gene silencing. We treated the CK2α-silenced Jeko-1 clone with different concentrations of Ibrutinib added 24 h before harvesting. The combination between Ibrutinib and CK2α silencing induced a significant increase of AV positive cells as compared to the single treatments, confirming the results obtained with CX-4945 (Figure 2E). As expected, in the control IPTG-treated Jeko-1 WT cells we did not observe increased apoptosis compared to Ibrutinib-only treated cells. The synergistic growth-inhibiting/apoptosis-inducing effect was confirmed also by cell cycle analysis. CK2α silencing combined with Ibrutinib resulted in a significant increase in the percentage of cells in sub-G1 phase (likely apoptotic) as compared to the single treatments. Additionally, the combined treatment caused also a decrease in the percentage of cells in G0/G1 and G2/M phases as compared to the single conditions, suggesting a proliferation arrest. As expected, the cell-cycle distribution of Jeko-1 WT cells (used as control) treated with IPTG and Ibrutinib did not show any difference, confirming the specificity of the silencing strategy (Figure 2F).

**FIGURE 3:**
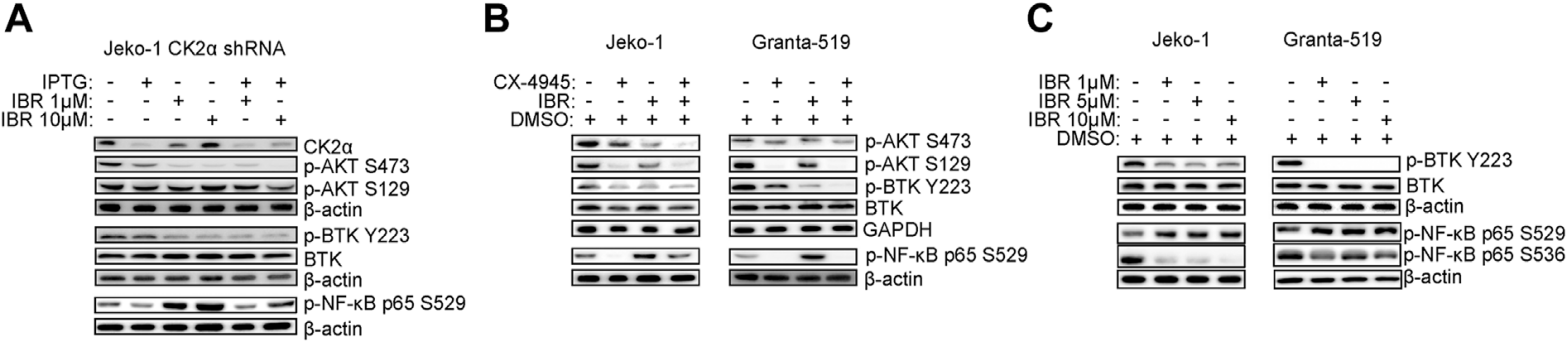
CK2 inhibition and Ibrutinib modulate BCR dependent signaling pathways. **(A,B)** Representative WB of PI3K/AKT, BTK and NF-κB dependent signaling in Jeko-1 CK2α shRNA cells after 6 days induction with IPTG 500 μM, 24 h treatment with IBR (1 and 10 μM) or their combination **(A)** and in Jeko-1 and Granta-519 cells **(B)** after 24 h treatment with DMSO (not higher than 0.1% v/v), CX4945 (1 μM for Jeko-1 and 2.5 μM for Granta-519), IBR (1 μM for each cell line) or their combination. **(C)** Representative WB showing the expression levels of phosphorylated BTK on Y223 (p-BTK Y223) and NF-κB p65 on S529 (p-NF-κB p65 S529) and on S536 (p-NF-κB p65 S536) in Jeko-1 and Granta-519 cells after 24 h treatment with increasing doses of IBR (ranging from 1 to 10 μM for each cell line). β-actin and GAPDH were used as loading control. Experiments were repeated at least three times for each cell line.

At the molecular level CK2α silencing or CX-4945 impinged on BCR dependent signaling cascades, strengthening the Ibrutinib-induced reduction of phosphorylated BTK on Y223 and AKT on S473 (Figures 3A, B and Supplementary Figure S3). Moreover, CK2 inactivation caused a marked decrease in phosphorylation of the CK2 targets AKT S129 and p65/RelA S529 (Figures 3A,B and Supplementary Figure S3). This latter, CK2 dependent phosphorylation, was surprisingly induced by Ibrutinib in a dose-dependent manner (Figure 3C and Supplementary Figure S3), indicating an Ibrutinib induced activation of CK2 which could represent a potential drawback effect possibly antagonizing the efficacy of the drug. The activity of Ibrutinib was confirmed by the reduction in phosphorylation of BTK in Y223 and p65/RelA in S536. (Figure 3C and Supplementary Figure S3).

### CK2 inactivation boosts Venetoclax-induced cytotoxicity in MCL cells

**FIGURE 4:**
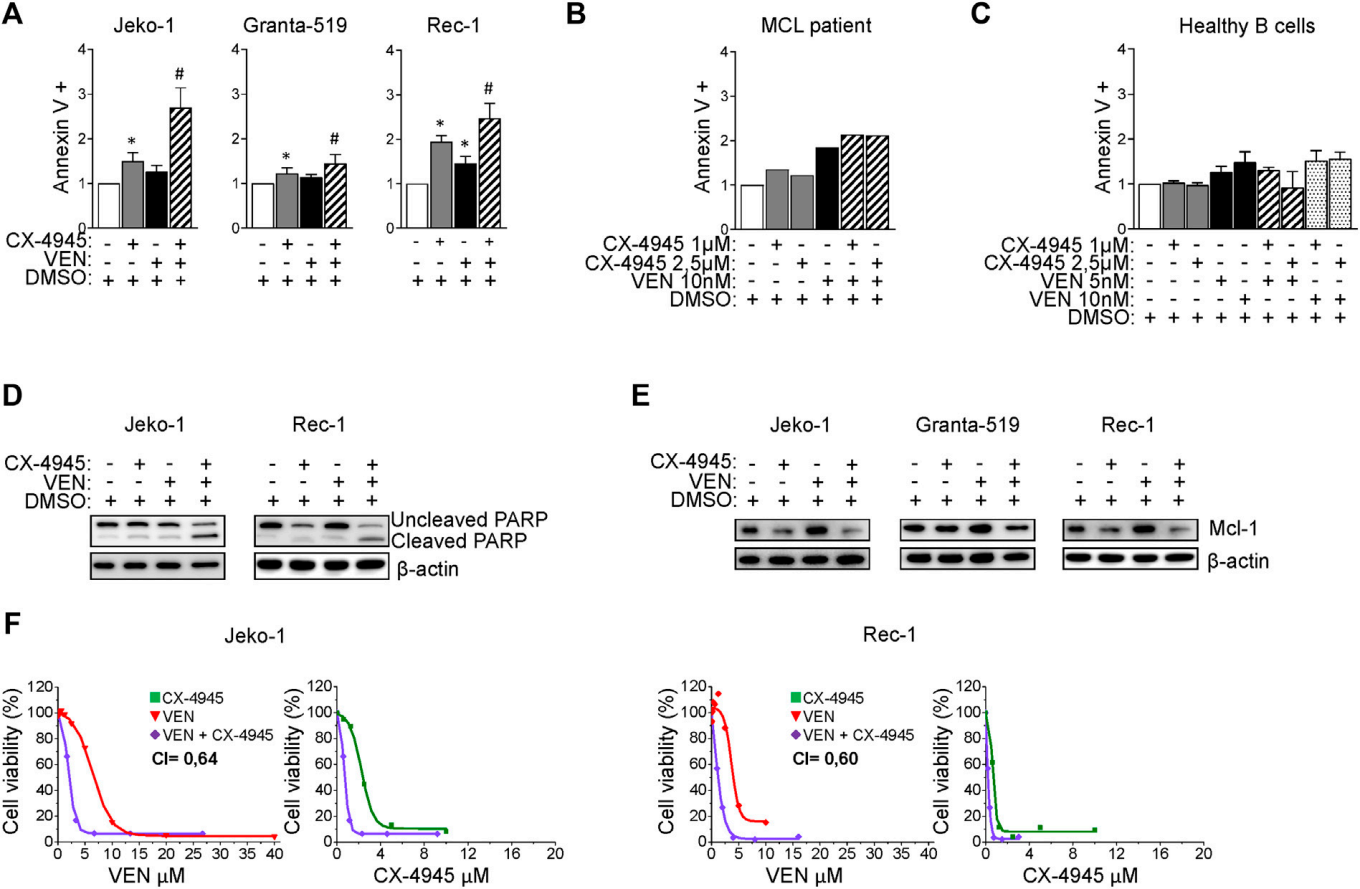
CX-4945 potentiates Venetoclax induced cytotoxicity. **(A)** Histograms depicting the percentage of Annexin V positive Jeko-1, Granta-519 and Rec-1 cells after 24 h treatment with DMSO (not higher than 0.1% v/v; white columns), CX-4945 (1 μM for Jeko-1 and Granta-519, 0.5 μM for Rec-1; grey columns), VEN (20 nM for Jeko-1, 1.25 nM for Granta-519 and 50 nM for Rec-1; black columns) or their combination (striped columns). Data are expressed as ratio over DMSO treated cells, mean ± SD of *n* = 10 (Jeko-1), *n* = 9 (Granta-519) and *n* = 6 (Rec-1) independent experiments. * indicates *p* < 0.05 compared to DMSO treated cells; # indicates *p* < 0.05 compared to both CX-4945 and VEN only treated cells. **(B)** Histogram showing the percentage of Annexin V positive B cells derived from one MCL patient after 24 h treatment with DMSO (not higher than 0.1% v/v; white column), CX-4945 (1 μM and 2.5 μM; grey columns), VEN (10 nM; black column) or their combination (striped columns). Data are expressed as ratio over DMSO treated cells. **(C)** Histogram showing the percentage of Annexin V positive healthy B cells treated for 24 h with DMSO (not higher 0.1% v/v; white column), CX-4945 (1 μM and 2.5 μM; grey columns), VEN (5 and 10 nM; black columns) or their combination (striped and dotted colums). Data are normalized on DMSO treated cells and are expressed as the mean ± SD of *n* = 3 independent experiments. **(D)** Representative WB showing the expression levels of uncleaved and cleaved PARP in Jeko-1 and Rec-1 cells treated as in **(A)**. β-actin was used as loading control. Experiments were repeated at least three times for each cell line. **(E)** Representative WB showing the expression levels of Mcl-1 in Jeko-1, Granta-519 and Rec-1 cells treated as in **(A)**. β-actin was used as loading control. Experiments were repeated at least three times for each cell line. **(F)** Dose response curves of Jeko-1 and Rec-1 cells incubated for 72 h with increasing concentrations of CX4945 (green curves), VEN (red curves) or their combination (purple curves). For Jeko-1, IC_50_ of the single treatments were 2.30 μM for CX4945 and 6.68 μM for VEN. IC_50_ for CX4945 used in combination with VEN was 0.74 μM, while IC_50_ for VEN used in association with CX-4945 was 2.13 μM. The calculated CI was 0.64. For Rec-1, IC_50_ of the single treatments were 0.76 μM for CX-4945 and 4.0 μM for VEN. IC_50_ for CX-4945 used in combination with VEN was 0.23 μM, while IC_50_ for VEN used in association with CX-4945 was 1.22 μM. The calculated CI was 0.60. Data are presented as averaged percentage over control of three independent experiments for each cell line.

Venetoclax is a BH3-mimetic already approved for the treatment of R/R Chronic Lymphocytic Leukemia and Acute Myeloid Leukemia and with clinical activity in MCL. However, also for this drug mechanisms of resistance may arise, including modulation of survival signaling cascades such as AKT (Pham et al., 2018) or increased expression of the anti-apoptotic Mcl-1 protein, buffering the inhibition of Bcl-2 (Prukova et al., 2019). Given the above-described results which proved that CK2 is a positive regulator of both AKT and the Bcl-2 family member Mcl-1 (Figure 1), we tested the effect of CK2 inactivation on Venetoclax-induced cytotoxicity. We first performed a dose response curve of Venetoclax in our MCL cell lines, treating them with increasing doses of Venetoclax for 24 h. We found that Granta-519 were the most sensitive while Jeko-1 and Rec-1 were less responsive to this drug (Supplementary Figure S4). Next, we analyzed the effects of combining CK2 and Bcl-2 inhibitors on MCL apoptosis by labelling cells with AV. CX-4945 potentiated Venetoclax-induced apoptosis in all the MCL cell lines tested and in B cells derived from one patient (Figures 4A, B), while it had no effects on healthy B cells (Figure 4C) where cooperation between CX-4945 and Venetoclax was not observed. The increased apoptosis in MCL cells was confirmed also by the immunoblot analysis of PARP cleavage, which was enhanced in the cells treated with the combination of Venetoclax and CX-4945 (Figure 4D and Supplementary Figure S5). Venetoclax treated MCL cells showed an upregulated expression of the pro-survival Bcl-2 family member Mcl-1 protein. Captivatingly, CX-4945 led to a substantial reduction of the Venetoclax-induced increase of Mcl-1, again potentially counteracting a deleterious Venetoclax-induced drawback effect (Figure 4E and Supplementary Figure S5).

Mcl-1 transcript level was unaffected in CX-4945 or Venetoclax treated cells (Supplementary Figure S6A), while its protein level was reduced in CK2 chemically inhibited cells, through a post-translational mechanism that seems to not involve caspase neither the proteasome (Supplementary Figure S6B).

**FIGURE 5:**
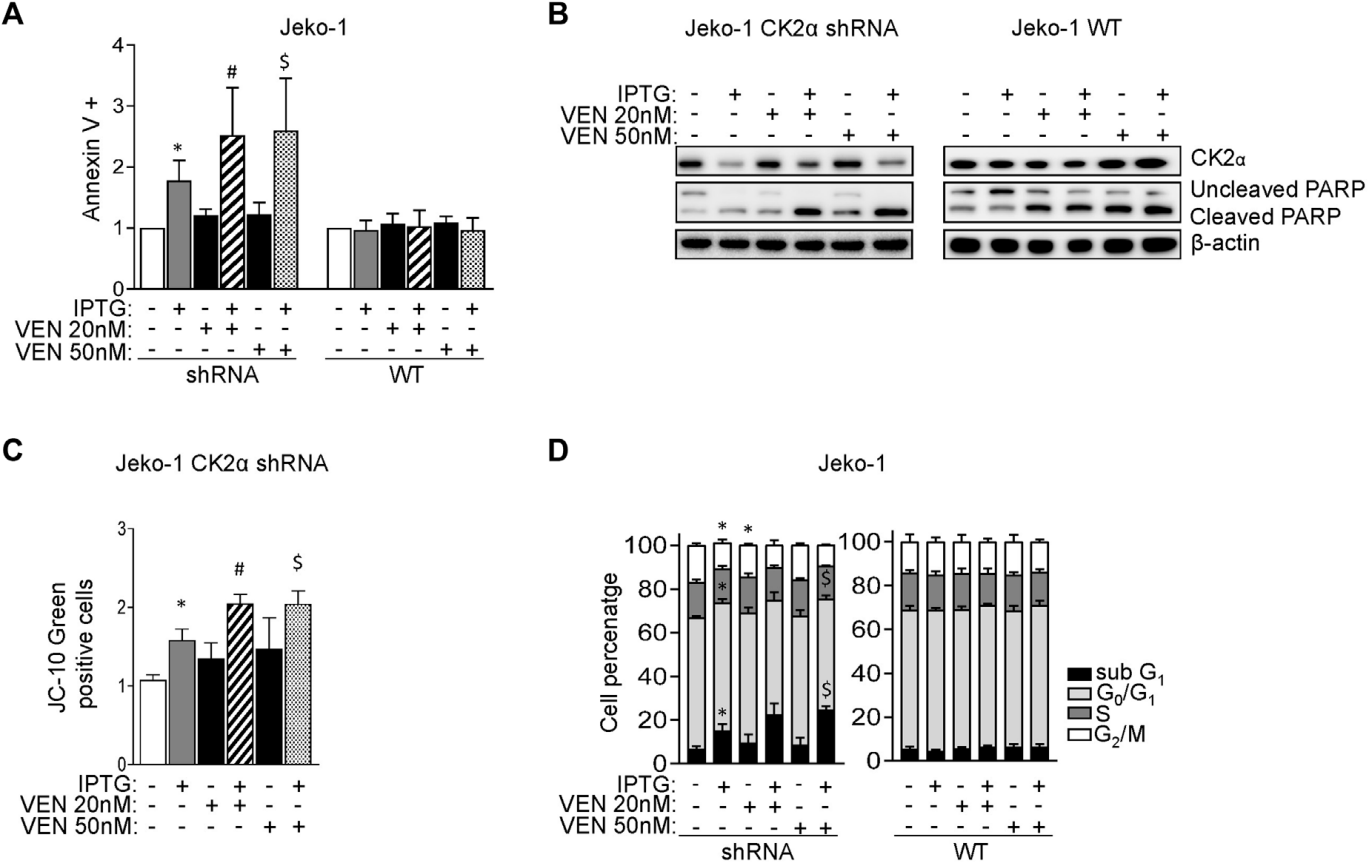
CK2α silencing potentiates the cytotoxic and cytostatic effects induced by Venetoclax. **(A)** Histogram showing the percentage of Annexin V positive Jeko-1 CK2α shRNA and Jeko-1 WT cells after 6 days of CK2α silencing with IPTG 500 μM (grey columns), 24 h treatment with VEN 20 and 50 nM (black columns) or their combination (striped and dotted columns). Data are expressed as ratio over untreated cells, mean ± SD of *n* = 12 independent experiments for each cell line. * indicates *p* < 0.05 compared to untreated cells; # indicates *p* < 0.05 compared to both IPTG and VEN 20 nM only treated cells; $ indicates *p* < 0.05 compared to both IPTG and VEN 50 nM only treated cells. **(B)** Representative WB showing the expression levels of uncleaved and cleaved PARP in Jeko-1 CK2α shRNA and Jeko-1 WT cells treated as in **(A)**. β-actin was used as loading control. Experiments were repeated at least three times for each cell line. **(C)** Histogram depicting the percentage of JC-10 green positive Jeko-1 CK2α shRNA cells treated as in **(A)**. Data are expressed as ratio over averaged untreated cells, mean ± SD of *n* = 3 independent experiments. * indicates *p* < 0.05 compared to untreated cells; # indicates *p* < 0.05 compared to both IPTG and VEN 20 nM only treated cells; $ indicates *p* < 0.05 compared to both IPTG and VEN 50 nM only treated cells. **(D)** Cell cycle distribution of Jeko-1 CK2α shRNA and Jeko-1 WT cells treated as in **(A)**. Histograms columns sections represent mean ± SD of *n* = 4 (Jeko-1 CK2α shRNA) and *n* = 6 (Jeko-1 WT) independent experiments of the sub G_1_ (black), G₀/G₁ (light grey), S (dark grey) and G₂/M (white) cell cycle phases. * indicates *p* < 0.05 compared to untreated cells; $ indicates *p* < 0.05 compared to both IPTG and VEN 50 nM only treated cells.

To confirm the possible synergism between CK2 and Bcl-2 inhibitions, we evaluate cell growth and calculated the CI between drugs. Remarkably, the CI between CX-4945 and Venetoclax was less than 1 even in the less Venetoclax-sensitive cell lines (Figure 4F) suggesting a synergic cytotoxic effect between CX-4945 and Venetoclax. To validate the results obtained with the CK2 chemical inhibitor and to exclude any off-target effects, we performed the same experiments using the IPTG inducible CK2α directed shRNA Jeko-1 clone. The same results were obtained upon CK2α gene silencing, as judged by the increased percentage of AV^+^ cells and extent of PARP cleavage (Figures 5A, B and Supplementary Figure S5) and JC-10 green^+^ cells (indicating mitochondrial membrane potential loss) (Figure 5C) in the combination compared to single treatments. Cell cycle analysis showed an increased percentage of apoptotic cells in the sub-G1 phase in CK2-silenced cells and a modulation of the GO/G1 and G2/M phases indicative of a deregulation of cell proliferation (Figure 5D).

## Discussion

Given the unfavourable prognosis of relapsed/refractory MCL, even in the era of novel drugs targeting cell signaling or of immunotherapy with Chimeric Antigen Receptor- T cells, the introduction of novel therapeutic approaches that might affect oncogenic survival signaling networks, is mandatory (Vose, 2017). In recent years, the pleiotropic S/T protein kinase CK2 has emerged as a potential target in solid and blood tumours (Strum et al., 2021). The overexpression of this kinase and its key role in the maintenance of the cancer phenotype have been reported in several haematological malignancies, among which MM (Manni et al., 2014), CLL (Martins et al., 2011) and B-NHL (Pizzi et al., 2015).

In accordance with the paradigm known as “non-oncogene addiction”, CK2 overexpression is believed to be responsible of propelling basic mechanisms of cell proliferation and survival (Strum et al., 2021). We previously demonstrated that CK2 is overexpressed in primary MCL samples compared to healthy ones and that treatment with CX-4945 caused not only apoptosis of MCL cell lines but also of patient derived MCL B cells (Manni et al., 2013).

In the present study we have provided strong evidence that protein kinase CK2 sustains active Bcl-2 family related and BCR signaling networks in MCL, by upregulating Mcl-1 expression and BTK, AKT and NF-κB activation. The results showed in our work highlight the function of CK2 in protecting MCL cells from Ibrutinib and Venetoclax-induced apoptosis, therefore indicating that CK2-targeting could be envisioned as a rational therapeutic strategy to improve anti-MCL Ibrutinib and Venetoclax-induced cytotoxicity.

Importantly, the evidence of this critical role of CK2 in MCL was obtained by a dual approach, by chemical inhibition of the kinase and knock down of its expression. Manipulating CK2 expression in MCL cells, we proved that CK2α, but not CK2β, sustains MCL survival and proliferation, *in vitro* in cultured cells and *in vivo* in a MCL xenograft mouse model. We showed that the anti-apoptotic effect of CK2 in MCL might depend on its role on different pro-growth signaling. CK2α silencing reduced anti-apoptotic markers expression such as Mcl-1 and Pro-caspase 3. CK2 also regulates the activating phosphorylation of three BCR downstream cascades, S129 and S473 AKT, S529 NF-κB p65 and Y223 BTK, all of them important for MCL clonal expansion. The significance of these pathways in MCL are well-described. Overactivation of NF-κB and PI3K/AKT is exploited in B cell tumors and in MCL (Rudelius et al., 2006; Balaji et al., 2018). Targeting BCR signaling with the BTK inhibitor Ibrutinib is an effective therapeutic option for R/R MCL cases. However, Ibrutinib resistance in MCL occurs and it may be attributable to mutations in BCR downstream elements, such as in the NF-κB pathway, BTK or PLCγ2 (Hershkovitz-Rokah et al., 2018).

We showed that CK2 sustains BCR related signaling events, namely at the level of NF-κB and PI3K/AKT activation. We also described an unanticipated role of CK2 in regulating BTK activity in cultured cells and *in vivo* in a MCL xenograft mouse model (Figures 1, 3)*.* How CK2 chemical inactivation or gene silencing may cause a reduction of the phosphorylation of BTK on Y223 remains to be elucidated. The possibility that CK2 physically interacts with BTK or phosphorylates BTK through another kinase is presently unknown and will deserve further investigation. Targeting CK2 along with Ibrutinib modulating BTK active phosphorylation, enhanced the cytostatic and cytotoxic effect of Ibrutinib, even in the Ibrutinib less-sensitive Granta-519 cells (Figure 2). Of note, we observed that Ibrutinib increased the CK2 dependent phosphorylation of p65/RelA on S529 (Figure 3) (which is known to fully activate p65/RelA), suggesting that Ibrutinib may cause the activation of CK2. How this p65/RelA S529 increased phosphorylation is mediated needs to be defined. We performed an *in silico* search which revealed that S529 might potentially be a target for Glycogen Synthase Kinase 3 (GSK3), which is a target of AKT/PI3K signaling cascade. AKT mediated phosphorylation of GSK3 on Ser 9, 21 inhibits GSK3 and stabilizes Myc transcription factors, supporting proliferation (Mancinelli et al., 2017). Therefore, there is also the possibility that Ibrutinib, by hampering AKT activity, induces S529 p65/RelA phosphorylation through GSK3. The upregulation of phosphorylated S529 p65/RelA could represent a potential drawback effect contributing to drug resistance. In support of our data, a recent work that employed a proteomic approach to identify global kinome changes due to Ibrutinib resistance reported that Ibrutinib resistant (IR) MCL cells undergo a kinome reprogramming. Among others, the PI3K/AKT dependent cascade and CK2α were found to be more active in IR cells compared to the parental one (Zhao et al., 2017). Collectively, this experimental evidence supports a role for CK2 over-activation in Ibrutinib resistance and suggests that this kinase may be a rational target in IR MCL cells.

In a therapeutic perspective, it can be envisioned the design of novel inhibitors, that may either target CK2 or may be dually specific against this and other BCR dependent kinases, as previously done (e.g the PI3Kδ/CK1ε inhibitor Umbralisib, which showed clinical activity in CLL and lymphomas (Davids et al., 2019)).

We have also demonstrated that CK2 inhibition may synergize with another strategy used in the treatment of MCL and other haematological malignancies, i.e. the targeting of Bcl-2, through the selective, oral inhibitor Venetoclax (Cang et al., 2015; Yalniz and Wierda, 2019). CK2 has been shown to sustain Bcl-2 related family members expression in MCL (Figure 1, Supplementary Figure S1 and (Manni et al., 2013)). Therefore, we reasoned that its inhibition would affect Venetoclax mechanism of action. Indeed, this approach resulted to be synergistic with Venetoclax in all the cell lines studied, even in those that displayed a lower sensitivity to Bcl-2 inhibition, such as Jeko-1 and Rec-1 (Pham et al., 2018; Prukova et al., 2019). Indeed, the combination of CX-4945 and Venetoclax demonstrated higher efficacy in these cell lines anticipating a potential strategy to overcome Venetoclax resistance. To this regard, it is noteworthy that CK2 inhibition caused a dramatic reduction of Mcl-1 protein levels in MCL cells (Figure 4). The mechanism by which CX-4945 causes Mcl-1 protein decrease in the tested MCL cell lines is still unclear since it does not seem to be caspase or proteasome dependent (Supplementary Figure S6). Mcl-1 translation in the cell is finely controlled and different miRNAs have been implicated in the control of Mcl-1 translation (as an example miR29 and other miRNAs have been identified to reduce Mcl-1 protein expression) (Mott et al., 2007; Lam et al., 2010). Moreover, other pathways such as eIF2α/stress response (Fritsch et al., 2007) or the mTORC1 (Mills et al., 2008) cascades have been implicated in the regulation of Mcl-1 translation. Therefore, the exact mechanism by which Mcl-1 protein is reduced upon CK2 inactivation is still unknown and will deserve further investigation. Mcl-1 is one of the major players involved in Venetoclax resistance and our data and others demonstrated that its expression levels could be influenced by CK2 (Manni et al., 2013; Lazaro-Navarro et al., 2021). The present work defined a synergy between CK2 inactivation and Venetoclax in inducing MCL cell death, confirming the evidence reported by others in Acute Lymphoblastic Leukemia (ALL) cells (Lazaro-Navarro et al., 2021). Remarkably, the selectivity of CX-4945 was also validated in the experiments of CK2α silencing, ascribing its apoptotic influence to CK2 inhibition and not to *off target* effects (Figure 5). In addition to its impact on Mcl-1, CK2 can modulate mitochondrial apoptosis through the regulation of BID, a pro-apoptotic protein belonging to the Bcl-2 family. It has been reported that BID can be phosphorylated by CK2 in the proximity of the caspase-8 recognition site (T58), leading to BID insensitivity to caspase-8 cleavage and delayed apoptosis (Desagher et al., 2001). CK2 inhibition could prevent this mechanism of apoptosis-resistance, allowing the cleavage of full-length BID, the translocation of the fragment obtained to the mitochondria and the subsequent cytochrome c release. The concomitant exposure to Venetoclax would provoke an impairment of the sequestration of BID by Bcl-2, allowing a higher number of pro-apoptotic proteins to be subjected to proteolysis with consequent boosting of apoptosis. Lastly, the impact of CK2 on NF-κB and PI3K/AKT pathways could also play a significant role in Venetoclax mechanism of action. Indeed, Venetoclax resistance mechanism may also rely on enhanced NF-kB and AKT signaling. It has been reported that constitutively active AKT could phosphorylate the IKKα subunit on T23, inducing IKK complex activation, IκB degradation and NFκB translocation into the nucleus in embryonic fibroblasts (Bai et al., 2009). Additionally, Jayappa *et al* suggested that the activation of the NF-*κ*B pathway in MCL cells could lead to an enhanced expression of the anti-apoptotic proteins Bcl-xL and Mcl-1, decreasing the dependency of tumor cells on Bcl-2 and thus their sensitivity to Venetoclax (Jayappa et al., 2017). Therefore, CK2 could be an essential node in the survival signaling network that could mediate Venetoclax induced resistance.

**FIGURE 6:**
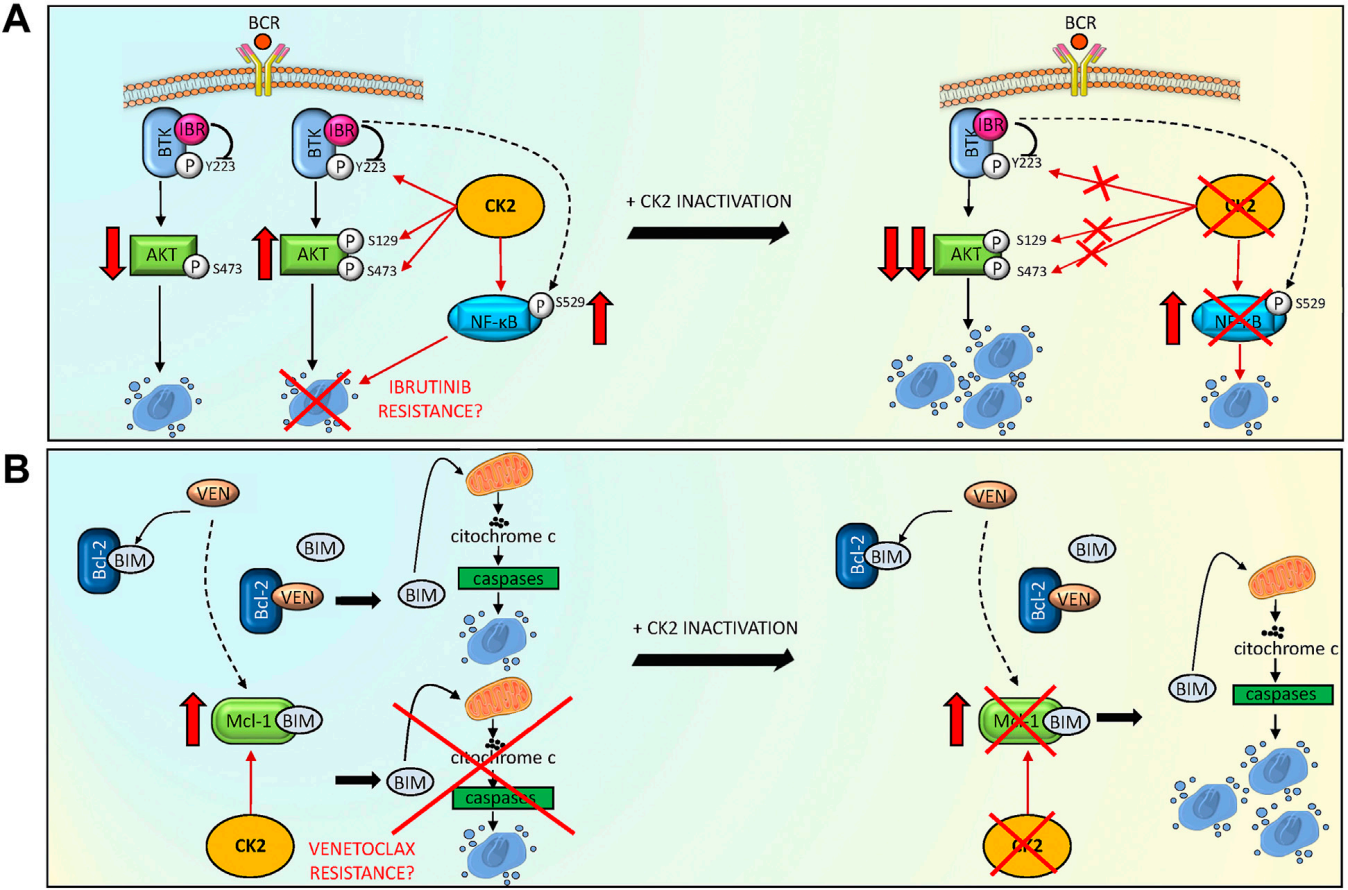
CK2 inactivation potentiates Ibrutinib and Venetoclax induced cytotoxicity, overcoming potential drug resistance mechanisms. **(A)** Model summarizing the role of CK2 inhibition to reduce the activation of MCL survival BCR dependent signaling pathways, potentiating Ibrutinib (IBR) induced cytotoxicity. Ibrutinib reduced BTK and AKT phosphorylation on Y223 and S473 respectively, causing MCL cell apoptosis. CK2 sustains the anti-apoptotic phosphorylation of BTK on Y223, of AKT on S129 and S473 (potentially contrasting the apoptosis capability of Ibrutinib) and of NF-κB on S529 (inducing a possible mechanism of resistance). The combination of CK2 inactivation and Ibrutinib potentiates the downregulation of these pro-survival signaling pathways, overcoming potential CK2 dependent mechanisms of resistance. **(B)** Model showing a possible mechanism adopted by CK2 inactivation to overcome a Venetoclax (VEN) induced mechanism of resistance. VEN binds to Bcl-2 releasing the pro-apoptotic BIM protein, causing cytochrome C/caspase mediated apoptosis. However, an increase in Mcl-1 anti-apoptotic protein can be observed, relieving VEN apoptosis inducing capability. The addiction of CK2 inactivation determines a reduction in Mcl-1 protein levels, overcoming the VEN induced mechanism of resistance, ultimately potentiating MCL cell death. The figure was made using Servier Medical art templates licensed under a Creative Common Attribution 3.0 Generic License. http://smart.servier.com/accessed on 25/03/2022.

In conclusion, our results demonstrated that CK2 sustains the activation of critical signaling pathways in MCL cells. CK2 could be a central connector in the survival signaling cascades that could interfere with Ibrutinib or Venetoclax-induced cytotoxicity (Figure 6). Its inactivation can potentiate, in a synergistic manner, the cytotoxicity induced by BTK and Bcl-2 inhibitors. We provided evidence that CK2 inactivation led to the downregulation of potential mechanism of resistance that may arise with Ibrutinib or Venetoclax, i.e. the activation of NF-kB and Mcl-1 as summarized in Figure 6. Our data support the further testing of CK2 inhibitors in MCL in combination therapies.

## Data Availability

The original contributions presented in the study are included in the article/Supplementary Material. Further inquiries can be directed to the corresponding authors.
