# Supplementary material for: Protein Kinase CK2 represents a new target to boost Ibrutinib and Venetoclax induced cytotoxicity in mantle cell lymphoma
Source: Front Cell Dev Biol. 2022 Aug 11;10:935023. doi: 10.3389/fcell.2022.935023 (PMC9403710; doi:10.3389/fcell.2022.935023)
Supplement: Supplementary file 1 [file DataSheet1.PDF]

Supplementary Material

Supplementary Figures

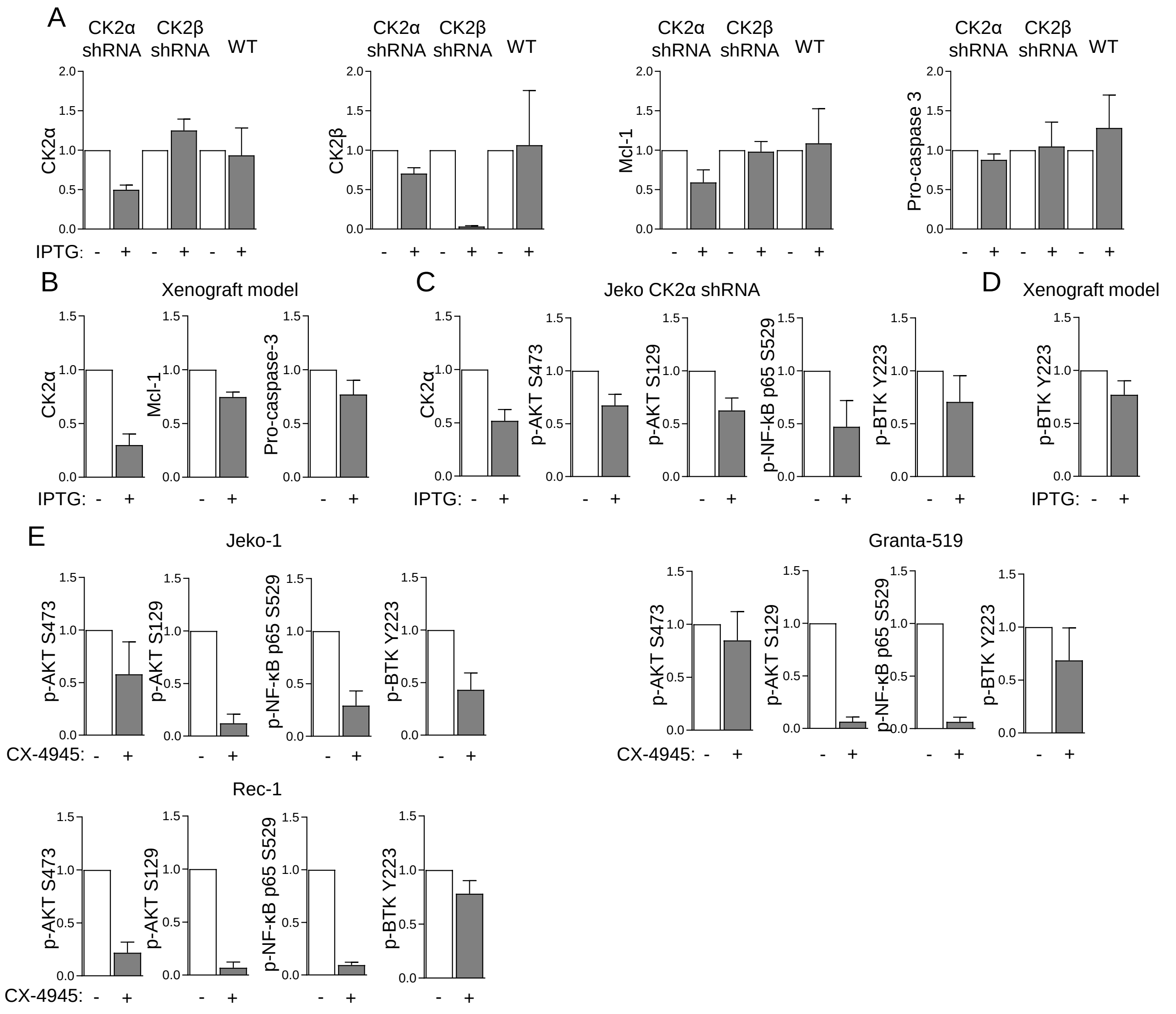

Supplementary Figure 1. CK2α sustains MCL cell growth and survival signaling pathways downstream the BCR. Western blot densitometric analysis of CK2α, CK2β, Mcl-1 and Pro-caspase 3 expression over β-actin in Jeko-1 CK2α shRNA, Jeko-1 CK2β shRNA and Jeko-1 WT cells after 6 days induction with IPTG 500 µM (A), and of CK2α, Mcl-1 and Pro-caspase-3 in CK2α shRNA cells xenografted in NOD SCID mice and treated *in vivo* with IPTG for 25 days (B). Densitometric analysis of CK2α, phosphorylated AKT on S473 (p-AKT S473) and on S129 (p-AKT S129), phosphorylated NF-κB-p65 on S529 (p-NF-κB-p65 S529), and BTKY223 phosphorylation (p-BTK Y223) over β-actin in Jeko-1 CK2α shRNA cells after 6 days induction with IPTG 500 µM (C) and BTKY223 phosphorylation over β-actin in Jeko-1 CK2α shRNA cells xenografted in mouse and treated with IPTG

as in B (D). (E) Densitometric analysis of p-NF-κB-p65 S529, p-AKT S129, p-AKT S473 and p-BTKY223 phosphorylation over β-actin in MCL cell lines Jeko-1, Granta-519 and Rec-1 after 24 h treatment with DMSO or CX-4945 (2,5 µM for Jeko-1 and Granta-519; 0,5 µM for Rec-1). Data represent mean densitometric values ± SD of protein of interest/β-actin normalized over untreated cells of at least 3 independent experiments. β-actin was used as loading control.

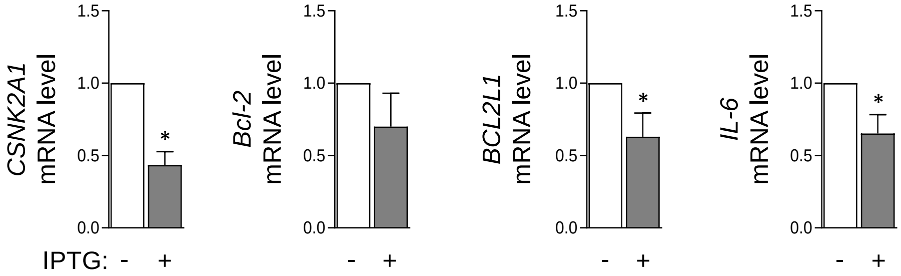

Supplementary Figure 2: CK2α gene silencing reduces NF-kB target gene expression. Quantitative Real Time PCR analysis of *CSNK2A1* and NF-kB targets mRNA expression (*BCL-2*, *BCL2L1*, *IL-6*), in Jeko-1 IPTG inducible CK2α directed shRNA clone treated with IPTG 500µM for 6 days. GAPDH was used as reference gene and values were normalized to the untreated condition. Data represent mean ± SD of at least 3 independent experiments. * indicates $p < 0.05$.

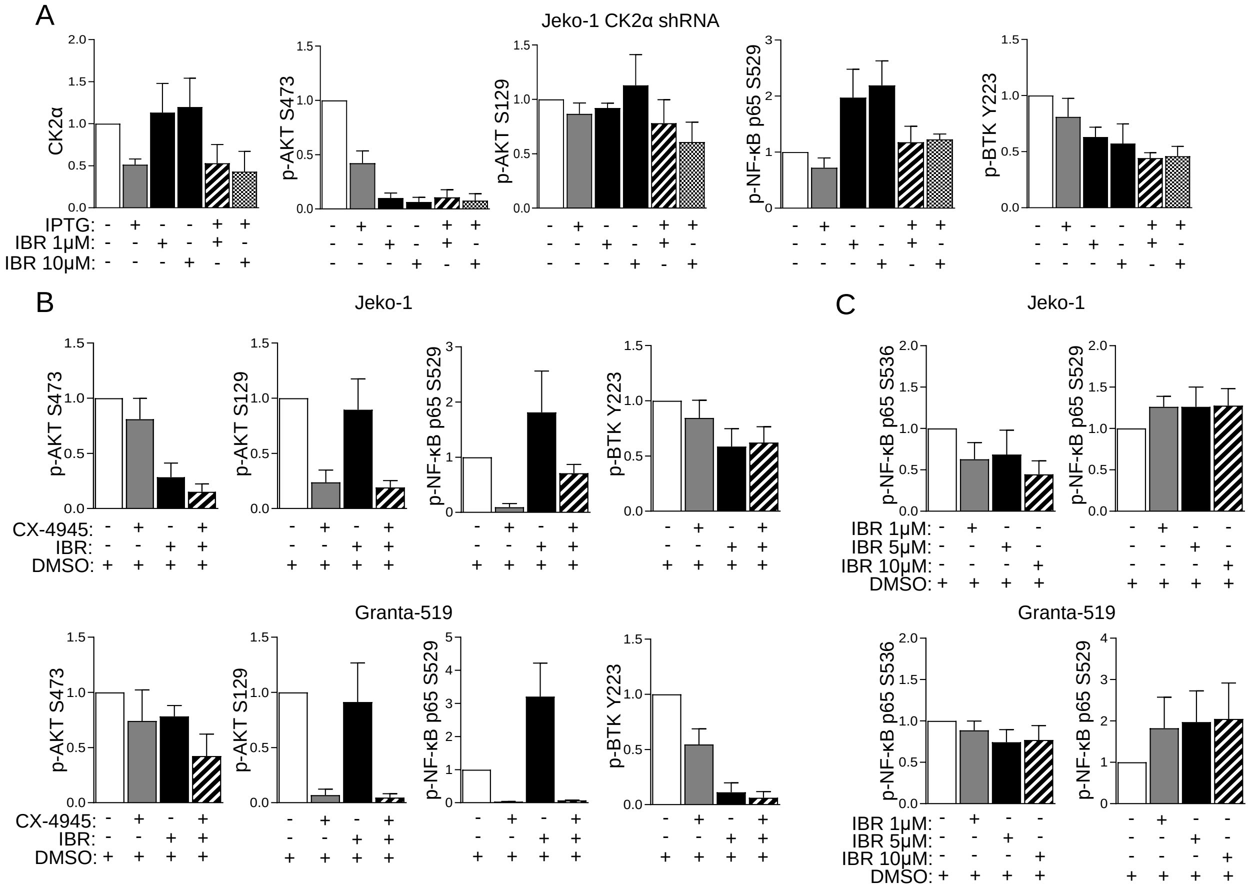

Supplementary Figure 3: CK2 inactivation and Ibrutinib modulate BCR dependent signaling pathways. (A) Western blot densitometric analysis of CK2α, AKT S473 and AKT S129 phosphorylation (p-AKT S473 and p-AKT S129), NF-κB-p65 phosphorylation on S529 (p-NF-κB-p65 S529), and BTKY223 phosphorylation (p-BTK Y223) over β-actin in Jeko-1 CK2α shRNA cells after 6 days induction with IPTG 500 μM, 24h treatment with IBR (1 μM and 10 μM) or their combination. (B) Densitometric analysis of p-AKT S473 and p-AKT S473, p-NF-κB-p65 S529, and p-BTK Y223 over GAPDH in Jeko-1 (upper panel) and Granta-519 cells (lower panel) after 24 h treatment with DMSO, CX4945 (1 μM for Jeko-1 and 2,5 μM for Granta-519), IBR (1 μM for each cell line) or their combination (B). (C) densitometric analysis of NF-κB p65 phosphorylation on S536 and on S529 (p-NF-κB-p65 S536 and p-NF-κB-p65 S529), over β-actin in Jeko-1 (upper panel) and Granta-519 cells (lower panel) after 24 h treatment with DMSO, IBR 1 μM, 5 μM, 10 μM for each cell line. In all panels data represent mean densitometric values ± SD of protein of interest/β-actin or GAPDH normalized over untreated cells or DMSO treated cells of at least 3 independent experiments. β-actin and GAPDH were used as loading control.

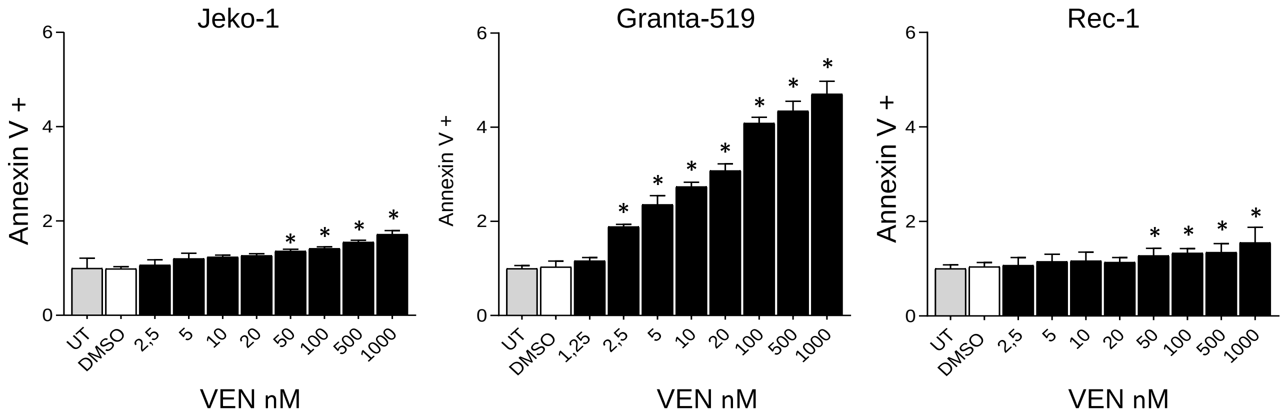

Supplementary Figure 4: Venetoclax causes MCL cell lines apoptosis. Histograms showing the percentage of Annexin V positive Jeko-1, Granta-519 and Rec-1 cells after 24 h treatment with DMSO (not higher than 0,1% v/v) or increasing doses of VEN (ranging from 2,5-1000 nM for Jeko-1 and Rec-1 and from 1,25-1000 nM for Granta-519). Data were normalized on the averaged untreated cells (UT) and expressed as mean ± SD of n=3 (Granta-519 and Jeko-1) and n=6 (Rec-1) independent experiments. * indicates $p<0,05$ compared to the untreated (UT) condition.

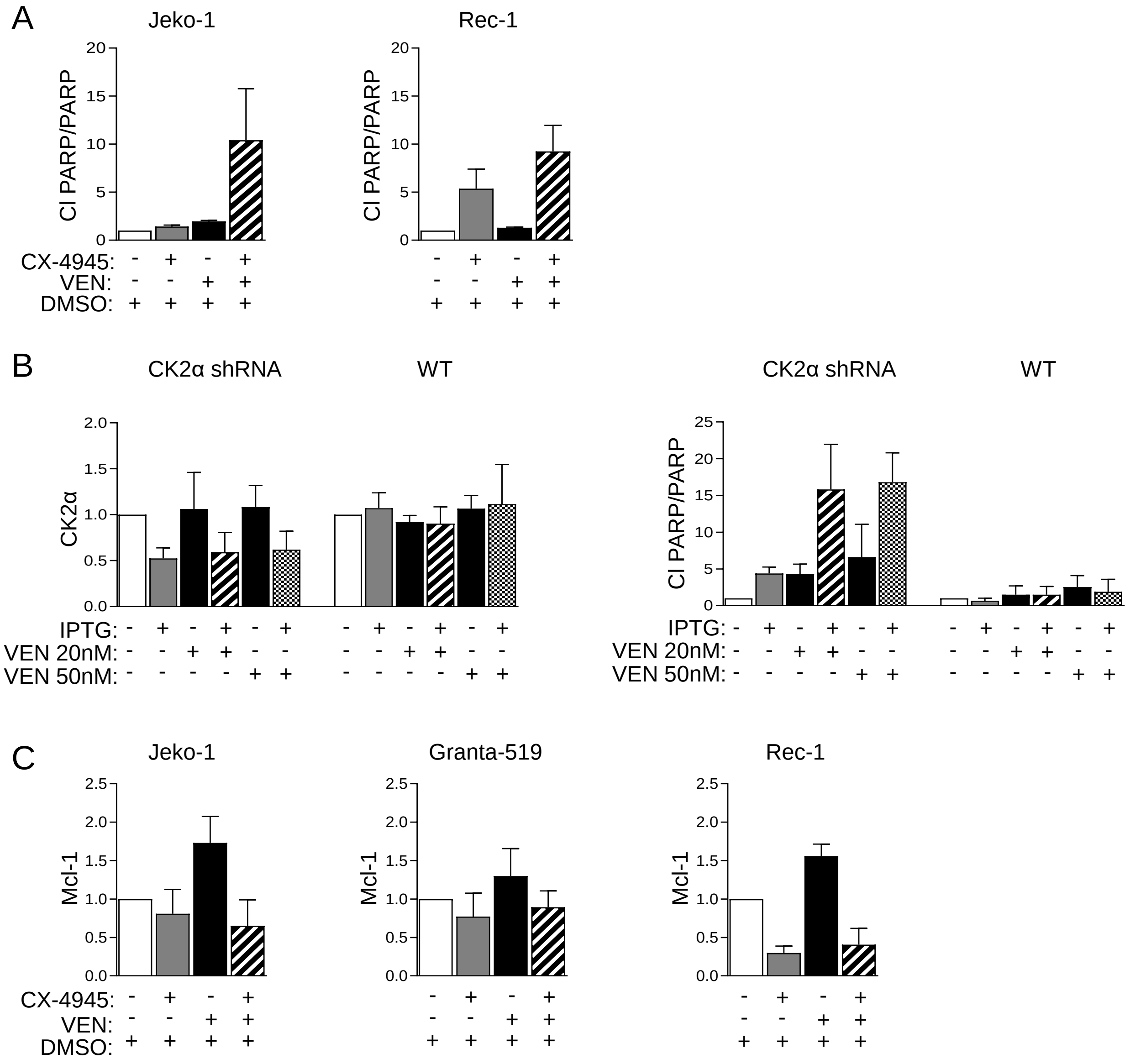

Supplementary Figure 5: CK2 inactivation potentiates Venetoclax induced cytotoxicity. Western blot densitometric analysis of the expression levels of cleaved PARP(Cl PALP)/uncleaved PARP in Jeko-1 and Rec-1 cells treated for 24h with DMSO, CX-4945 (1 µM for Jeko-1; 0,5 µM for Rec-1), VEN (20 nM for Jeko-1 and 50 nM for Rec-1) or the combination of CX-4945 and VEN (A) and in Jeko-1 CK2α shRNA and Jeko-1 WT cells after 6 days of CK2α silencing with IPTG 500µM, 24 h treatment with VEN 20 nM and 50 nM or their combination (B). CK2α expression was also evaluated in B. (**C**) Western blot densitometric analysis of the expression levels of Mcl-1 in Jeko-1, Granta-519 and Rec-1 cells treated with DMSO, CX-4945 (1 µM for Jeko-1 and Granta-519, 0,5 µM for Rec-1) VEN (20 nM for Jeko-1, 1,25 nM for Granta-519 and 50 nM for Rec-1) or their combination. In all panels, data represent mean densitometric values ± SD of protein of interest/β-actin normalized over

untreated or DMSO treated cells of at least 3 independent experiments. β-actin was used as loading control.

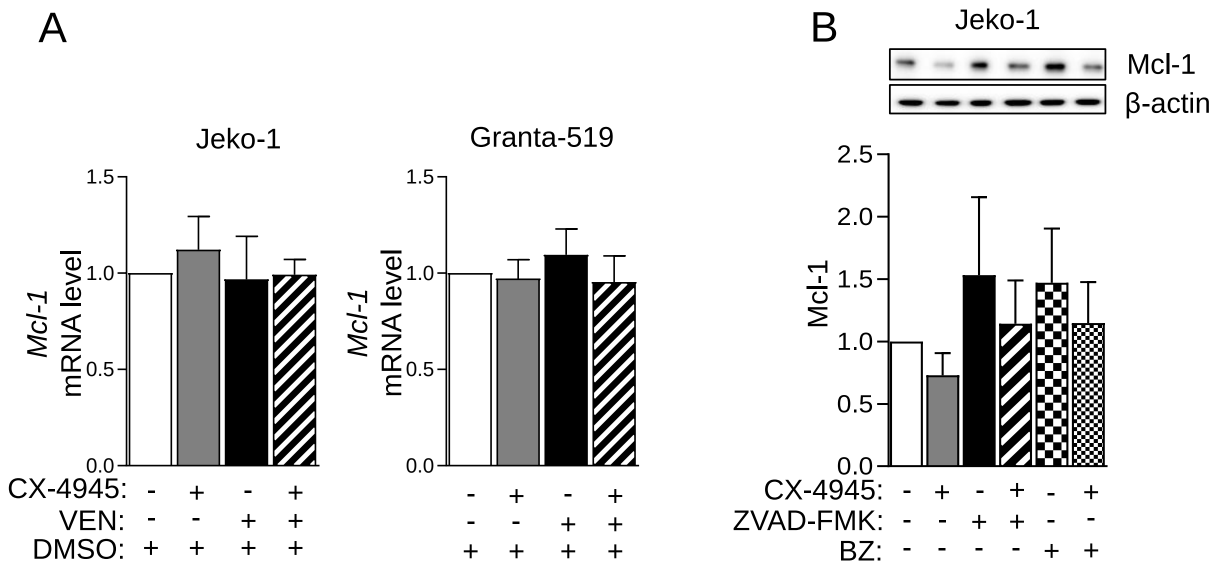

Supplementary Figure 6. CK2 chemical inhibition reduces Mcl-1 total protein level through a post-translational mechanism. (A) Treatment of Jeko-1 (left panel) or Granta 519 (right panel) cells with the CK2α chemical inhibitor CX-4945 or Venetoclax does not change *Mcl-1* mRNA expression**.** Quantitative Real Time PCR analysis of *Mcl-1* mRNA expression performed in Jeko-1 and Granta-519 treated with CX-4945 1µM, Venetoclax 20nM (for Jeko-1) or CX-4945 1µM, Venetoclax 1.25 nM (for Granta-519) or the combination of the two compounds for 24h. Data represent mean ± SD of 6 independent experiments in Jeko-1 and 4 independent experiments in Granta-519 cells. GAPDH was used as reference gene. (B) Representative WB of Mcl-1 protein expression in Jeko-1 cells treated with CX-4945 1µM, alone, or in association with Z-VAD-FMK 2µM or bortezomib (BZ) 5nM for 24h. β-actin was used as loading control. Bottom panel represents densitometry analysis of the bands and data are expressed as mean densitometric values ± SD of Mcl-1/β-actin normalized over untreated cells of 4 independent experiments.
